# Association between mitral annulus calcification and severity of coronary artery disease assessed by SYNTAX score in patients presented with acute coronary syndrome

**DOI:** 10.3389/fcvm.2024.1413984

**Published:** 2024-10-18

**Authors:** Afsaneh Esmailpour, Soroush Nematollahi, Reza Hali, Mohammad Sadeghian, Sepehr Nayebirad, Ahmad Vakili

**Affiliations:** ^1^ Tehran Heart Center, Cardiovascular Diseases Research Institute, Tehran University of Medical Sciences, Tehran, Iran; ^2^ Department of Epidemiology and Biostatistics, School of Public Health, Tehran University of Medical Sciences, Tehran, Iran

**Keywords:** mitral annulus calcification, SYNTAX score, coronary artery disease, acute coronary syndrome, angiography

## Abstract

**Objectives:**

Mitral annulus calcification (MAC) has been linked to cardiovascular disease severity, but its relationship with the SYNTAX score (SS) in acute coronary syndrome (ACS) patients remains unclear. This study aimed to investigate the association between MAC and SS in ACS patients to explore the role of MAC in predicting cardiovascular disease severity.

**Methods:**

We conducted a cross-sectional study of 233 ACS patients at Tehran Heart Center, Tehran, Iran, from December 2021 to August 2022. Patients with prior coronary artery disease (CAD) were excluded. Demographic data, risk factors, and medical history were extracted from clinical files. SS was determined using coronary angiography, and MAC was assessed via two-dimensional transthoracic echocardiography.

**Results:**

The study population had a mean age of 58.79 years, with 74.7% male. MAC was present in 24.9% of participants, and 57% of those with MAC had an SS above 23. Univariate analysis revealed a significant association between MAC and higher SSs (odds ratio: 1.84, 95% CI: 1.02–3.39; *P* = 0.046). However, multivariable analysis showed that only left ventricular ejection fraction (LVEF) was independently associated with SS (odds ratio: 0.94, 95% CI: 0.89–0.99; *P* = 0.015).

**Conclusion:**

While MAC was initially associated with higher SSs in ACS patients, only LVEF emerged as an independent predictor in the multivariable analysis. Although MAC may not be independently associated with SS, it may serve as a useful echocardiographic indicator of more severe CAD in ACS.

## 1 Introduction

Mitral annulus calcification (MAC) is a chronic, degenerative condition that worsens over time (1, 2). The etiology of MAC involves multiple factors, including atherosclerosis-like mechanisms that trigger the transformation of specific cells in the heart valves (3, 4). The degree of calcification can vary from a noticeable spicule to a large chip-like mass, usually at the back of the posterior cusp. Mitral valve calcification can occur as a ring-shaped deposit or along leaflets. Occasionally, it can even increase the length of the anterior leaflet (5). MAC is often asymptomatic and is detected incidentally during transthoracic echocardiography (TTE) (6). It predominantly affects women and individuals over 70 (7).

The SYNTAX score (SS) is a tool used to assess the severity of coronary artery involvement (8). It is a quantitative anatomical scoring system that evaluates the extent of coronary artery involvement based on angiographic obstructive lesions (8, 9). The number, location, degree, type, and complexity of lesions are considered when calculating the scores. This scoring system helps clinicians determine whether coronary artery disease patients require revascularization and which revascularization method is most appropriate (10). The American and European Revascularization Guidelines have approved this scoring system as a beneficial tool for managing CAD patients (8, 10, 11).

Previous studies have suggested a connection between MAC and various cardiovascular conditions, such as atrial and ventricular enlargement, myocardial infarction, atrial fibrillation, and cerebrovascular accidents (1, 2, 7, 12–16). While some studies have indicated a potential link between MAC and SS (17), others have found no such association (18). The exact relationship between MAC and SS remains unclear, and further investigation is necessary to determine whether MAC can predict CAD severity. The primary objective of this study is to assess the correlation between MAC and SS, with the ultimate goal of determining whether MAC can be used to predict the severity of CAD based on the SYNTAX score.

## 2 Methods and materials

### 2.1 Population

This cross-sectional study, conducted from December 2021 to August 2022 at the Tehran Heart Center in Iran, aimed to investigate the relationship between MAC and SS in patients with ACS. We enrolled all patients with a diagnosis of ACS who were consecutively admitted to the hospital. At the same time, those with a history of coronary artery bypass grafting (CABG) or percutaneous coronary intervention (PCI) were excluded to avoid prior intervention influence. The study has been approved by the Research Ethics Committees of the Tehran Heart Center on November 4, 2021 (IR.TUMS.THC.REC.1400. 059).

### 2.2 Design and instruments

We collected demographic features, risk factors (such as hypertension, diabetes, dyslipidemia, and smoking), and past medical and surgical history from the clinical records of patients who had been diagnosed with ACS. Additionally, we obtained patients’ laboratory values from their medical records at the time of admission. An expert interventional cardiologist calculated each patient's SS using coronary angiography. A qualified echocardiologist with a fellowship degree in advanced echocardiography, blinded to the angiography results, performed transthoracic echocardiography on all patients using the Philips Healthcare Affinity 70c system with an S1–5 transducer. The MAC was identified as a dense echocardiographic signal at the junction of the atrioventricular groove and the posterior mitral leaflet. We divided the patients into two groups based on their SS, with a 23 cutoff point (17). We then compared demographic, clinical, and echocardiographic parameters between these groups.

### 2.3 Statistical analysis and sample size

We presented quantitative variables using the mean ± standard deviation for normally distributed variables and the median with interquartile range for the non-normally distributed variables. We check the normality of the distributions by visually inspecting histograms and conducting descriptive analysis alongside the Kolmogorov‒Smirnov test. Categorical variables were summarized as frequencies and percentages. We compared quantitative variables using the independent samples *t*-test. Nonparametric tests were applied for non-normally distributed variables. Categorical variables were compared using the chi-square test. We analyzed univariate logistic regression to assess potential relationships between the variables and the SS. We performed a multiple logistic regression analysis to examine the possible confounding effect of the predictors. *P*-values less than 0.05 were considered significant. We used the R statistical language (version 4.3.2) (19) and the packages ResourceSelection (version 0.3.6) (20), ggstatsplot (version 0.12.1) (21), and dplyr (version 1.1.4) (22).

## 3 Results

We enrolled a total of 267 patients in the study. Patients who did not mention a history of PCI but had prior stenting observed during angiography, as well as patients who did not consent to CAG and left our center before the angiography, were excluded. We included 233 patients with an average age of 58.79 ± 11.65 years. The study comprised 174 males (74.7%) and 59 females (25.3%). The angiographic analysis found that 201 out of 233 patients (86.3%) had CAD. Although initial evaluation in the emergency department suggested a possible ACS, subsequent diagnostic workup, including coronary angiography, revealed that 32 patients did not have underlying CAD, with alternative diagnoses such as myocarditis being established instead. According to the SS classification, 106 patients had an SS greater than 23.

A significant association was observed between an SS above 23 and the prevalence of diabetes, with 44 diabetic patients in this group compared to 30 in the lower SS group (*P* = 0.003). The distribution of ACS subtypes was as follows: ST-elevation myocardial infarction (STEMI) occurred in 132 patients, non-ST-elevation myocardial infarction (*N*STEMI) in 60 patients, and unstable angina (UA) in 41 patients.

Notably, patients with high SSs exhibited significantly larger left ventricular end-systolic diameters (LVESDs) compared to those with lower SSs (38 mm vs. 37 mm, *P* = 0.013). Furthermore, the left ventricular ejection fraction (LVEF) was significantly reduced in patients with high SSs (*P* < 0.001). MAC was observed in 24.9% of participants, with a significantly higher prevalence among patients with SSs above 23 (31%, *P* = 0.044). A comparison of baseline characteristics and medical history, including hypertension, hyperlipidemia, smoking status, triglyceride levels, body mass index (BMI), and cholesterol levels, revealed no significant differences between patients with high and low SSs. The participants’ demographic, clinical, and echocardiographic characteristics are detailed in Tables 1, 2.

**Table 1:** Demographic and clinical characteristics of the participants based on the SS^a^.

Characteristic	Overall, *N* = 233^b^	<23, *N* = 127^b^	≥23, *N* = 106^b^	*p*-value^c^
Demographics				
Male gender	174 (74.7%)	92 (72.4%)	82 (77.4%)	0.390
Age	58.79 (11.65)	56.54 (12.05)	61.49 (10.59)	**0**.**002**
BMI	27.65 (4.21)	27.83 (4.35)	27.43 (4.04)	0.688
BSA	1.88 (0.19)	1.88 (0.20)	1.87 (0.16)	0.833
Medical history				
Cigarette smoking	76 (32.6%)	37 (29.1%)	39 (36.8%)	0.214
Opium consumption	36 (15.5%)	19 (15.0%)	17 (16.0%)	0.821
Diabetes	74 (31.8%)	30 (23.6%)	44 (41.5%)	**0**.**003**
HTN	102 (43.8%)	51 (40.2%)	51 (48.1%)	0.223
HLP	112 (48.1%)	58 (45.7%)	54 (50.9%)	0.422
Positive family history	41 (17.6%)	26 (20.5%)	15 (14.2%)	0.207
History of CHF	3 (1.3%)	–	–	–
History of CVA	1 (0.4%)	–	–	–
History of renal failure	2 (0.9%)	–	–	–
History of PVD	1 (0.4%)	–	–	–
Laboratory findings				
Hb	14.50 (1.66)	14.57 (1.68)	14.43 (1.63)	0.733
Cr, *Median (IQR)*	1.0 (0.8, 1.1)	0.9 (0.8, 1.1)	1.0 (0.9, 1.2)	0.108
FBS, *Median (IQR)*	107.0 (92.0, 146.0)	102.0 (89.0, 125.5)	116.0 (99.0, 156.5)	**<0**.**001**
TG, *Median (IQR)*	124.0 (85.0, 180.0)	125.0 (84.0, 177.0)	121.5 (85.3, 182.8)	0.713
TCH	162.81 (37.58)	158.91 (36.70)	167.48 (38.26)	0.075
HDL, *Median (IQR)*	39.0 (32.0, 46.0)	39.0 (33.0, 46.0)	39.0 (32.0, 46.0)	0.634
LDL	96.39 (29.54)	93.55 (28.01)	99.78 (31.05)	0.113

Bold values were statistically significant (*p* < 0.05).

^a^ SS cutoff = 23.

^b^ *n* (%); Mean (SD).

^c^ Wilcoxon rank sum test; Pearson's Chi-squared test; Fisher's exact test; independent *t*-test. SS, SYNTAX score; BMI, body mass index; BSR, body surface area,; HTN, hypertension; HLP, hyperlipidemia; CHF, congestive heart failure; CVA, cerebrovascular accident; PVD, peripheral vascular disease; Hb, hemoglobin; Cr, creatinine; FBS, fasting blood sugar; TG, triglyceride; TCH, total cholesterol; HDL, high-density cholesterol; LDL, low-density cholesterol.

**Table 2:** Cardiac and echocardiography parameters in patients based on the SS^a^.

Parameter	Overall, *N* = 233^b^	<23, *N* = 127^b^	≥23, *N* = 106^b^	*p*-value^c^
ECG rhythm				
NSR	230 (99%)	–	–	–
AF-AFL	3 (1.3%)	–	–	–
MAC				**0**.**044**
Without MAC	175 (75.1%)	102 (80.3%)	73 (68.9%)	
MAC	58 (24.9%)	25 (19.7%)	33 (31.1%)	
Angiography result				**<0**.**001**
Non-coronary	32 (13.7%)	32 (25.2%)	0 (0.0%)	
Coronary	201 (86.3%)	95 (74.8%)	106 (100.0%)	
Angiography reason				**0**.**005**
STEMI	132 (56.7%)	60 (47.2%)	72 (67.9%)	
NSTEMI	60 (25.8%)	38 (29.9%)	22 (20.8%)	
UA	41 (17.6%)	29 (22.8%)	12 (11.3%)	
Echocardiographic parameters				
LA diameter, *Median (IQR)*	39.0 (36.0, 42.0)	39.0 (36.0, 42.0)	39.0 (36.0, 42.0)	0.906
LA area, *Median (IQR)*	20.0 (18.0, 22.0)	21.0 (18.0, 23.0)	20.0 (18.0, 22.0)	0.475
LA volume, *Median (IQR)*	62.0 (51.0, 72.0)	62.0 (52.0, 73.0)	62.0 (50.0, 71.0)	0.681
LA volume indexed, *Median (IQR)*	34.0 (28.0, 38.0)	34.0 (28.0, 37.5)	34.0 (28.0, 38.0)	0.990
LVEDD, *Median (IQR)*	50.0 (46.0, 53.0)	50.0 (47.0, 52.0)	50.0 (46.0, 53.0)	0.271
LVESD, *Median (IQR)*	37.0 (33.0, 40.0)	37.0 (32.0, 39.0)	38.0 (34.0, 42.0)	**0**.**013**
LVEF, *Median (IQR)*	47.5 (40.0, 52.5)	50.0 (42.5, 52.5)	42.5 (33.1, 50.0)	**<0**.**001**
É septal, *Median (IQR)*	6.0 (5.0, 7.0)	6.0 (5.0, 7.0)	6.0 (4.3, 7.0)	**0**.**003**
É lateral, *Median (IQR)*	8.0 (7.0, 9.0)	8.0 (7.0, 10.0)	7.0 (6.0, 9.0)	**0**.**002**
E velocity, *Median (IQR)*	60.0 (50.0, 75.0)	60.0 (50.0, 72.5)	64.5 (50.0, 75.0)	0.652
TAPSE, *Median (IQR)*	19.0 (18.0, 22.0)	19.0 (18.0, 22.0)	19.0 (17.0, 21.0)	0.523
RVSm, *Median (IQR)*	11.0 (10.0, 12.0)	11.0 (10.0, 12.0)	11.0 (10.0, 12.0)	0.474
Aortic atherosclerosis	52 (22.3%)	27 (21.3%)	25 (23.6%)	0.671
Aortic calcification	31 (13.3%)	13 (10.2%)	18 (17.0%)	0.131
LVH	21 (9.0%)	9 (7.1%)	12 (11.3%)	0.261

Bold values were statistically significant (*p* < 0.05).

^a^ SS cutoff = 23.

^b^ *n* (%); Mean (SD).

^c^ Wilcoxon rank sum test; Pearson's Chi-squared test; Fisher's exact test; independent *t*-test. SS, SYNTAX score; ECG, electrocardiography; NSR, normal sinus rhythm; AF, atrial fibrillation; AFL, atrial flutter; MAC, mitral annulus calcification; STEMI, ST-elevation myocardial infarction; NSTEMI, non-ST-elevation myocardial infarction; UA, unstable angina; LA, left atrium; LVEDD, left ventricular end-diastolic diameter; LVESD, left ventricular end-systolic diameter; LVEF, left ventricular ejection fraction; TAPSE, tricuspid annular plane systolic excursion; RVSm, right ventricular peak systolic myocardial velocity; LVH, left ventricular hypertrophy.

Initial univariate logistic regression revealed that diabetes (OR: 2.29, 95% CI: 1.31–4.06, *P* = 0.004) and age (OR: 1.04, 95% CI: 1.02–1.07, *P* = 0.002) were associated with higher SSs. In contrast, LVEF was inversely associated with the SS (OR: 0.93, 95% CI: 0.90–0.96, *P* < 0.001). MAC was a significant predictor of SS (OR: 1.84, 95% CI: 1.02–3.39, *P* = 0.046). However, upon multivariable analysis, only LVEF maintained a significant association with the SS (OR: 0.94, 95% CI: 0.89–0.99, *P* = 0.015), while other factors did not demonstrate significant predictive value. The results of the logistic regression analysis are presented in Table 3.

**Table 3:** The relationships between statistically significant variables, as shown in Tables 1, 2, and the SS according to logistic regression analysis.

	Univariate			Multivariable			
Characteristic	OR	95% CI	*p*-value	OR	95% CI	*p*-value	GVIF
MAC	1.84	1.02, 3.39	**0**.**046**	1.28	0.64, 2.57	0.491	1.2
Diabetes	2.29	1.31, 4.06	**0**.**004**	1.77	0.95, 3.32	0.074	1.1
Angiography reason						0.227	1.1
STEMI	–	–	–	–	–	–	–
NSTEMI	0.48	0.25, 0.90	**0**.**023**	0.67	0.33, 1.34		
UA	0.34	0.16, 0.72	**0**.**006**	0.51	0.22, 1.17		
Age	1.04	1.02, 1.07	**0**.**002**	1.03	0.99, 1.06	0.129	1.6
LVESD	1.06	1.02, 1.11	**0**.**007**	0.99	0.92, 1.06	0.702	2.6
LVEF	0.93	0.90, 0.96	**<0**.**001**	0.94	0.89, 0.99	**0**.**015**	2.8
É septal	0.78	0.65, 0.92	**0**.**004**	1.05	0.81, 1.36	0.712	2.1
É lateral	0.83	0.74, 0.93	**0**.**002**	0.97	0.81, 1.14	0.693	2.0

SS, SYNTAX score; OR, odds ratio; CI, confidence interval; GVIF, generalized variance inflation factor; MAC, mitral annulus calcification; STEMI, ST-elevation myocardial infarction; NSTEMI, non-ST-elevation myocardial infarction; UA, unstable angina; LVESD, left ventricular end-systolic diameter; LVEF, left ventricular ejection fraction.

Bold values were statistically significant (*p* < 0.05).

**Figure 1:**
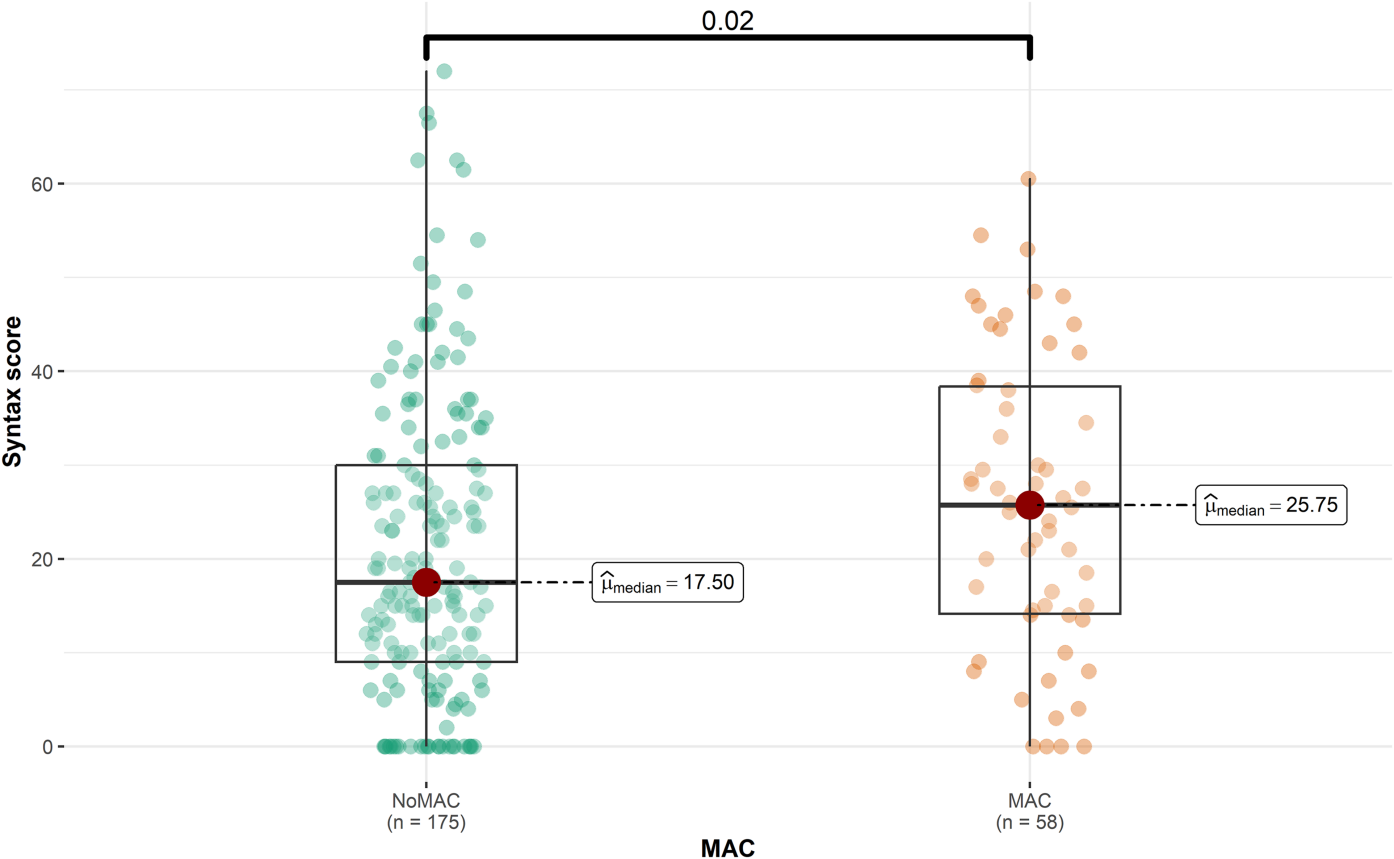
SYNTAX score and the presence of the MAC (mitral annulus calcification) (*P* = 0.020).

The majority of the participants (75.1%) did not have MAC. Patients with MAC had a significantly higher mean age than to those without MAC (65.86 vs. 56.45, *P* < 0.001). Furthermore, the prevalence of hypertension (HTN) and hyperlipidemia (HLP) was significantly higher among patients with MAC (60.3% and 62.1%, respectively) compared to those without MAC (*P* = 0.005 and *P* = 0.014, respectively). Figure 1 represents that the median SS was significantly higher in patients with MAC (25.8 vs. 17.5, *P* = 0.020), while the median LVEF was lower in this group (45 vs. 47.5, *P* = 0.041). Detailed patient information for the MAC and non-MAC groups is provided in Supplementary Table 1.

## 4 Discussion

This study examined the relationship between the SS and echocardiographic and non-echocardiographic parameters, focusing on MAC in the context of ACS. We categorized patients into high-SS and low-SS groups based on a cutoff of 23 (17). Our univariate analysis revealed a significant association between MAC and higher SSs (OR: 1.84). Additionally, both diabetes and age were significantly associated with an increase in SS (OR: 2.29 and 1.04, respectively). Echocardiographic features such as higher septal and lateral tissue velocities (É septal, É lateral), greater LVESD, and greater LVEF appeared protective against higher SSs. Following multivariable analysis, LVEF emerged as the sole independent predictor for SS (OR: 0.94). In contrast to the univariate analysis, MAC, diabetes, and age did not significantly influence the SS. The inverse relationship between LVEF and SS supports the idea that a higher SS indicates more extensive myocardial damage and reduced LVEF.

Previous studies have demonstrated a strong link between MAC and cardiovascular diseases. For instance, Fox et al. (12) found that MAC was associated with an increased risk of incident cardiovascular disease and all-cause mortality. Similarly, Potpara et al. (23) reported that MAC was linked to increased cardiovascular mortality and morbidity in patients with atrial fibrillation (23). Furthermore, Kohsaka et al. (7) observed that MAC significantly increased the risk of cardiovascular events, particularly ischemic stroke and vascular death, in a cohort of 1,955 patients. Notably, their analysis revealed that MAC was associated with increased rates of myocardial infarction (adjusted hazard ratio: 1.75). These findings collectively suggest that MAC significantly predicts adverse cardiovascular outcomes.

In addition, prior studies have shown an association between MAC and CAD (1, 2, 24). Kim et al. (25) reported a positive linear relationship between aortic valve calcification, MAC, and the incidence of CAD in the Korean population (25). However, in their case-control study, Nair et al. (26) found no significant differences in CAD incidence between MAC and non-MAC patients (26). In contrast, Atar et al. (1) observed that patients with MAC had a higher incidence of severe coronary artery disease, defined as stenosis of more than 70% in at least one major epicardial coronary artery or more than 50% in the left main coronary artery. The prevalence of severe coronary artery disease was significantly higher in MAC patients (88%) compared to those with a non-calcified mitral annulus (68%). Furthermore, MAC was found to have a positive predictive value of 92% for CAD (1). The underlying mechanisms driving the relationship between MAC and CAD likely involve shared pathophysiological processes, including chronic inflammation, endothelial dysfunction, lipid infiltration, joint atherosclerosis, and calcific degenerative processes (2, 5, 12).

While the SS is widely used and recommended for risk stratification of CAD, it has limitations. One remarkable issue is its binary classification of coronary dominance as either right or left, which oversimplifies the complexity of coronary artery anatomy. Additionally, the SS combines scores from lesions and adverse characteristics to derive the total score, but these elements differ significantly in their impact and weighting. This method can lead to inconsistencies in assessing the actual severity of CAD. Emerging angiographic scoring systems, such as the CatLet scoring system, address these limitations more effectively by providing a more nuanced evaluation of coronary anatomy and lesion characteristics (27, 28).

Despite numerous investigations into the potential link between MAC and CAD, evidence regarding this possible connection is scarce. Additionally, previous findings could be more consistent. For instance, Bhatt et al. (18) found no significant association between MAC and lesions with greater than 70% stenosis, the number of obstructive vessels, lesions with 50%–70% stenosis, or SS in their 2015 study. In contrast, Cerit et al. (17) reported that the presence of diabetes, active tobacco use, and MAC were significantly associated with higher SSs (odds ratio: 2.18, 1.96, and 1.76, respectively) in their multivariate analysis.

Our study suggests that there may be a connection between MAC and SS, as indicated by the univariate analysis. However, after adjusting for other factors in our multivariable analysis, including age, smoking, gender, and diabetes, the association between MAC and SS was no longer significant. This finding implies that the initial association between MAC and SS was likely influenced by other factors rather than a direct relationship. Nevertheless, MAC may be utilized as an indicator to identify patients with higher SS. Future studies are needed to confirm our findings.

### 4.1 Limitations

This study has a few limitations. As a cross-sectional study, it is limited in establishing causality relationships and can only assess prevalence and associations. Furthermore, like other observational studies, confounding factors may influence the results despite our efforts to minimize this effect through multivariable analysis.

## 5 Conclusion

In conclusion, our study revealed an initial association between MAC and higher SSs, but only LVEF emerged as an independent predictor in the multivariable analysis. Although MAC may not be independently associated with SS, it may still serve as a useful echocardiographic indicator of more severe CAD. This finding suggests that MAC may have potential as a marker for identifying patients with higher SSs, warranting further investigation.

## Data Availability

The original contributions presented in the study are included in the article/Supplementary Material, further inquiries can be directed to the corresponding author.
