# Supplementary material for: Association between mitral annulus calcification and severity of coronary artery disease assessed by SYNTAX score in patients presented with acute coronary syndrome
Source: Front Cardiovasc Med. 2024 Oct 18;11:1413984. doi: 10.3389/fcvm.2024.1413984 (PMC11527603; doi:10.3389/fcvm.2024.1413984)
Supplement: Supplementary file 1 [file Table1.docx]

**Supplementary Table 1**. Demographics and clinical characteristics of the participants based on the presence of MAC

| **Characteristic** | **Overall**  N = 233^†^ | **Without MAC**  N = 175^†^ | **MAC**  N = 58^†^ | **p-value**^‡^ |
| --- | --- | --- | --- | --- |
| **Demographics and history** |  |  |  |  |
| Age | 58.79 (11.65) | 56.45 (11.31) | 65.86 (9.69) | **<0.001** |
| Male gender | 174 (74.7%) | 135 (77.1%) | 39 (67.2%) | 0.133 |
| BMI | 27.65 (4.21) | 27.71 (4.22) | 27.48 (4.19) | 0.417 |
| Cigarette smoking | 76 (32.6%) | 62 (35.4%) | 14 (24.1%) | 0.112 |
| Opium consumption | 36 (15.5%) | 30 (17.1%) | 6 (10.3%) | 0.214 |
| Diabetes | 74 (31.8%) | 52 (29.7%) | 22 (37.9%) | 0.244 |
| HTN | 102 (43.8%) | 67 (38.3%) | 35 (60.3%) | **0.003** |
| HLP | 112 (48.1%) | 76 (43.4%) | 36 (62.1%) | **0.014** |
| Positive family history | 41 (17.6%) | 34 (19.4%) | 7 (12.1%) | 0.202 |
| History of CHF | 3 (1.3%) | 0 (0%) | 3 (5.2%) | **-** |
| History of CVA | 1 (0.4%) | 1 (0.6%) | 0 (0%) | **-** |
| History of renal failure | 2 (0.9%) | 1 (0.6%) | 1 (1.7%) | **-** |
| History of PVD | 1 (0.4%) | 1 (0.6%) | 0 (0%) | **-** |
| **Laboratory findings** |  |  |  |  |
| Hb | 14.50 (1.66) | 14.61 (1.61) | 14.19 (1.78) | 0.114 |
| Cr, *Median (IQR)* | 1.0 (0.8, 1.1) | 1.0 (0.8, 1.1) | 1.0 (0.8, 1.2) | 0.828 |
| FBS, *Median (IQR)* | 107.0 (92.0, 146.0) | 105.0 (91.0, 138.5) | 113.0 (98.3, 151.0) | 0.152 |
| TG, *Median (IQR)* | 124.0 (85.0, 180.0) | 121.0 (85.0, 188.5) | 131.0 (84.3, 156.3) | 0.644 |
| TCH | 162.81 (37.58) | 162.67 (38.28) | 163.24 (35.71) | 0.784 |
| HDL, *Median (IQR)* | 39.0 (32.0, 46.0) | 39.0 (32.0, 45.0) | 39.5 (32.3, 47.8) | 0.819 |
| LDL | 96.39 (29.54) | 95.88 (30.36) | 97.91 (27.08) | 0.573 |
| **Cardiac parameters** |  |  |  |  |
| ECG Rhythm |  |  |  | **-** |
| *NSR* | 230 (99%) | 172 (98%) | 58 (100%) |  |
| *AF-AFL* | 3 (1.3%) | 3 (1.7%) | 0 (0%) |  |
| **Angiography Result** |  |  |  | 0.192 |
| *Non-Coronary* | 32 (13.7%) | 27 (15.4%) | 5 (8.6%) |  |
| *Coronary* | 201 (86.3%) | 148 (84.6%) | 53 (91.4%) |  |
| SS, *Median (IQR)* | 19.0 (10.0, 32.5) | 17.5 (9.0, 30.0) | 25.8 (14.1, 38.4) | **0.020** |
| **Angiography reason** |  |  |  | 0.630 |
| *STEMI* | 132 (56.7%) | 96 (54.9%) | 36 (62.1%) |  |
| *NSTEMI* | 60 (25.8%) | 47 (26.9%) | 13 (22.4%) |  |
| *UA* | 41 (17.6%) | 32 (18.3%) | 9 (15.5%) |  |
| **Echocardiographic parameters** |  |  |  |  |
| LA diameter, *Median (IQR)* | 39.0 (36.0, 42.0) | 39.0 (36.0, 41.0) | 39.0 (36.0, 44.0) | 0.325 |
| LA area, *Median (IQR)* | 20.0 (18.0, 22.0) | 20.0 (17.0, 22.0) | 21.0 (18.0, 23.0) | 0.103 |
| LA volume, *Median (IQR)* | 62.0 (51.0, 72.0) | 62.0 (51.0, 72.0) | 63.0 (53.4, 72.4) | 0.668 |
| LA volume indexed, *Median (IQR)* | 34.0 (28.0, 38.0) | 34.0 (27.3, 37.0) | 35.0 (30.0, 38.0) | 0.101 |
| LVEDD, *Median (IQR)* | 50.0 (46.0, 53.0) | 50.0 (46.0, 53.0) | 50.0 (47.0, 52.8) | 0.505 |
| LVESD, *Median (IQR)* | 37.0 (33.0, 40.0) | 37.0 (32.0, 40.0) | 37.0 (34.0, 40.8) | 0.291 |
| LVEF, *Median (IQR)* | 47.5 (40.0, 52.5) | 47.5 (40.0, 52.5) | 45.0 (35.0, 50.0) | **0.041** |
| É septal, *Median (IQR)* | 6.0 (5.0, 7.0) | 6.0 (5.0, 7.0) | 5.0 (4.0, 6.0) | **<0.001** |
| É lateral, *Median (IQR)* | 8.0 (7.0, 9.0) | 8.0 (7.0, 10.0) | 7.0 (6.0, 9.0) | **0.008** |
| E velocity, *Median (IQR)* | 60.0 (50.0, 75.0) | 60.0 (50.0, 74.0) | 64.5 (50.3, 81.0) | 0.239 |
| TAPSE, *Median (IQR)* | 19.0 (18.0, 22.0) | 19.0 (18.0, 22.0) | 19.0 (18.0, 21.0) | 0.502 |
| RVSm, *Median (IQR)* | 11.0 (10.0, 12.0) | 11.0 (10.0, 12.0) | 11.0 (10.0, 12.0) | 0.994 |
| ^†^n (%); Mean (SD), ^‡^Pearson's Chi-squared test; Wilcoxon rank sum test; independent t-test. MAC: mitral annulus calcification, BMI: body mass index, HTN: hypertension, HLP: hyperlipidemia, CHF: congestive heart failure, CVA: cerebrovascular accident, PVD: peripheral vascular disease, Hb: hemoglobin, Cr: Creatinine, FBS: fasting blood sugar, TG: triglyceride, TCH: total cholesterol, HDL: high-density cholesterol, LDL: low-density cholesterol, ECG: electrocardiography, NSR: normal sinus rhythm, AF: atrial fibrillation, AFL: atrial flutter, SYNTAX: synergy between percutaneous coronary intervention with Taxus and cardiac surgery, STEMI: ST-elevation myocardial infarction, NSTEMI: non-ST-elevation myocardial infarction, UA: unstable angina, LA: left atrium, LVEDD: left ventricular end-diastolic diameter, LVESD: left ventricular end-systolic diameter, LVEF: left ventricular ejection fraction, TAPSE: tricuspid annular plane systolic excursion, RVSm: right ventricular peak systolic myocardial velocity, LVH: left ventricular hypertrophy. | | | | |
